# PPARγ sumoylation-mediated lipid accumulation in lung cancer

**DOI:** 10.18632/oncotarget.19700

**Published:** 2017-07-31

**Authors:** Ai N.H. Phan, Vu T.A. Vo, Tuyen N.M. Hua, Min-Kyu Kim, Se-Young Jo, Jong-Whan Choi, Hyun-Won Kim, Jaekyoung Son, Young-Ah Suh, Yangsik Jeong

**Affiliations:** ^1^ Department of Biochemistry, Wonju College of Medicine, Yonsei University, Wonju, Gangwon-do, Republic of Korea; ^2^ Department of Global Medical Science, Institute of Lifestyle Medicine, Wonju College of Medicine, Yonsei University, Wonju, Gangwon-do, Republic of Korea; ^3^ Asan Institute for Life Sciences, Asan Medical Center, College of Medicine, University of Ulsan, Seoul, Songpa-gu, Republic of Korea

**Keywords:** PPARγ, sumoylation, lipid metabolism, lung cancer

## Abstract

Metabolic reprogramming as a crucial emerging hallmark of cancer is critical for tumor cells to maintain cellular bioenergetics, biosynthesis and reduction/oxidation (REDOX) balance. Peroxisome proliferator-activated receptor gamma (PPARγ) is a nuclear hormone receptor regulating transcription of diverse gene sets involved in inflammation, metabolism, and suppressing tumor growth. Thiazolidinediones (TZDs), as selective PPARγ ligands, are insulin-sensitizing drugs widely prescribed for type 2 diabetic patients in the clinic. Here, we report that sumoylation of PPARγ couples lipid metabolism to tumor suppressive function of the receptor in lung cancer. We found that ligand activation of PPARγ dramatically induced *de novo* lipid synthesis as well as fatty acid beta (β)-oxidation in lung cancer both *in vitro* and *in vivo*. More importantly, it turns out that PPARγ regulation of lipid metabolism was dependent on sumoylation of PPARγ. Further biochemical analysis revealed that PPARγ-mediated lipid synthesis depletes nicotinamide adenine dinucleotide phosphate (NADPH), consequently resulting in increased mitochondrial reactive oxygen species (ROS) level that subsequently disrupted REDOX balance in lung cancer. Therefore, liganded PPARγ sumoylation is not only critical for cellular lipid metabolism but also induces oxidative stress that contributes to tumor suppressive function of PPARγ. This study provides an important insight of future translational and clinical research into targeting PPARγ regulation of lipid metabolism in lung cancer patients accompanying type 2 diabetes.

## INTRODUCTION

Lung cancer is the leading cause of cancer death with nearly 1.8 million diagnoses and 1.6 million deaths worldwide every year [1]. Most patients diagnosed with advanced lung cancer accompany poor prognosis, and thus standard therapies such as radio-chemotherapy or targeted treatment confer little benefit for the survival rate [2, 3].Thus, recently, different oncologic features between patients even with the same histologic type of tumors have led to personalized genomic screening to identify the most effective, individualized treatment option that matches each patient [4]. Although increasing advances in diagnosis and standard therapy have been achieved, treatment and prevention of lung cancer are unsatisfactory that requires a better understanding of the molecular mechanisms for the cancer progression. While oncogenic activation and/or inhibition of tumor suppressors are necessary to trigger cellular transformation for cancer development, metabolic reprogramming has become a crucial emerging hallmark of cancer in cancer initiation as well as progression [5, 6]. As a consequence of Warburg effect or aerobic glycolysis, cancer cells typically increase their reliance on glucose, glutamine, acetate, and other extracellular nutrients to maintain bioenergetics, biosynthesis, and redox balance [5, 7]. Importantly, to maintain tumor proliferation and overcome microenvironment oxidative stress, cancer cells utilize REDOX balance machinery to optimally control ROS production and reducing power NADPH level [8]. Therefore, increasing tumor vulnerability by targeting cancer metabolism, especially REDOX balance would become a potential adjuvant scheme to improve therapeutic efficacy for currently available anticancer treatment options in the clinic [9, 10].

PPARγ is a member of the nuclear receptor superfamily regulating lipid and glucose metabolism and has been widely studied for tumor associated cellular function in various cancers such as breast, colorectal, and lung cancer [11–17]. TZDs are PPARγ selective ligands sensitizing type 2 diabetic patients for insulin treatment and, of particular interest, clinical utilization of the drugs is clearly associated with better clinical outcomes in breast or lung cancer patients accompanying type 2 diabetes compared to the nonuser cancer patients [18–20]. Likewise, TZD treatment suppresses proliferation of primary cells derived from human liposarcoma and non-small cell lung cancer (NSCLC) growth in a PPARγ expression dependent manner [15, 16, 21, 22]. This implicates that biochemical feature of PPARγ regulating nutrient metabolism might be associated with its cell biological function as a tumor suppressor. Interestingly, a recent study showed that PPARγ-mediated growth inhibitory effect is associated with increased ROS generation, potentially due to induced mitochondrial β-oxidation of fatty acid [13]. This study suggests modulation of lipid metabolism could be an important biochemical feature for tumor suppressive function of PPARγ. Ligand-mediated PPARγ activity is further modulated by posttranslational modifications including phosphorylation, acetylation, ubiquitination, and sumoylation [11]. Amongst these, sumoylation of PPARγ2 at lysine 107 and lysine 395 is critical for ligand-dependent PPARγ activity to inhibit expression of inflammatory genes by stabilizing NcoR-corepressor complexes [23, 24]. In addition, FGF21 regulation of PPARγ sumoylation is involved in lipid accumulation in adipose tissues [25]. However, function of sumoylated PPARγ in cancer lipid metabolism and cell growth regulation remains unclear.

Here, we found that ligand-mediated PPARγ activation induces intracellular lipid accumulation via uptake as well as biosynthesis, rather than mitochondrial β-oxidation in lung cancer. Mechanistically, we demonstrated that sumoylation of PPARγ is necessary for the receptor-mediated intracellular lipid biosynthesis, leading to cellular NADPH reduction and thus ROS increase, which may contribute to tumor suppressive function of PPARγ. This study provides an insight of TZDs into regulating cancer lipid metabolism and further utilizing as a candidate adjuvant for anti-cancer therapy, especially for type 2 diabetes cancer patients.

## RESULTS

### PPARγ activation induces *de novo* lipid synthesis in lung cancer cells

**Figure 1:**
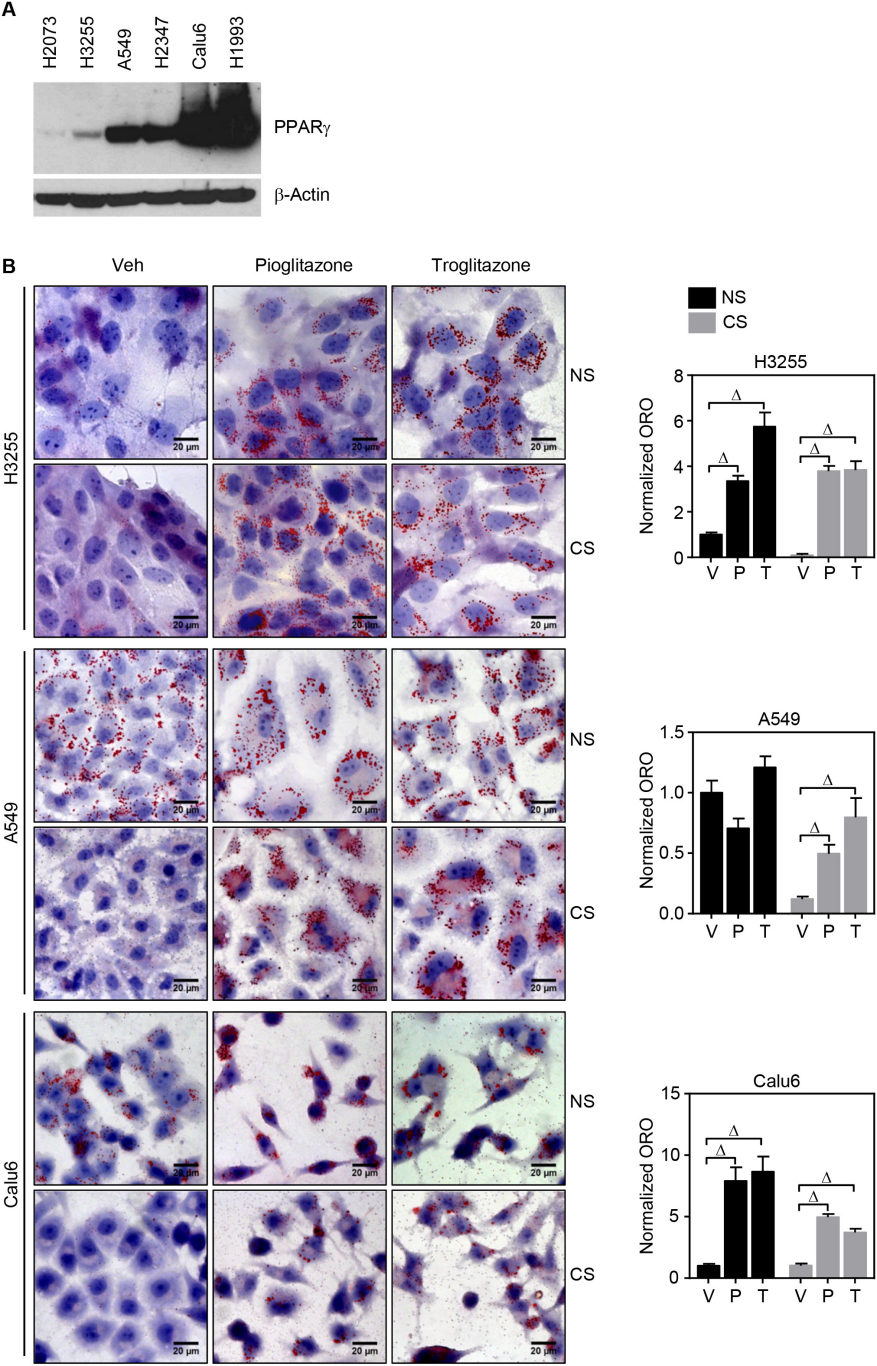

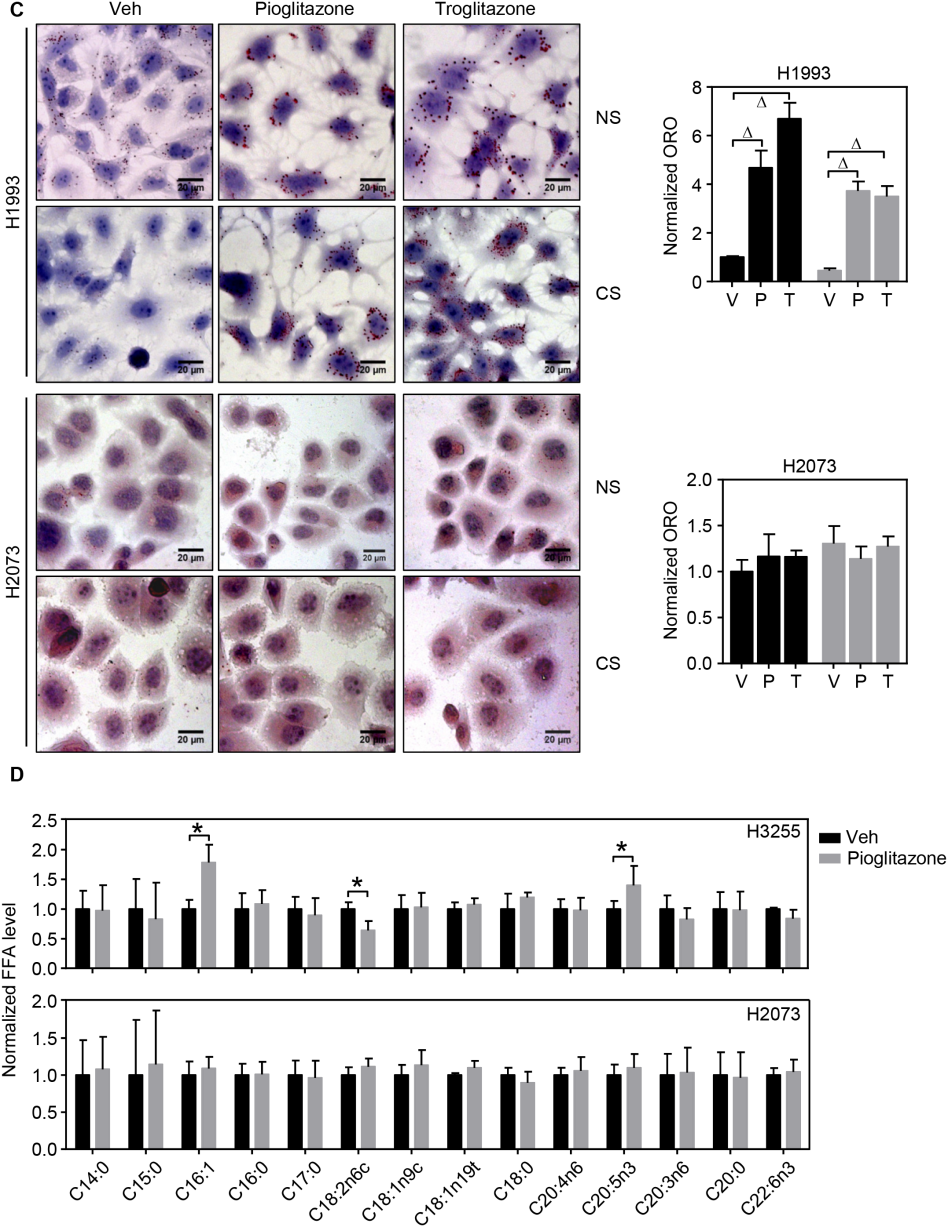
TZD treatment induces *de novo* lipid synthesis in lung cancer cells **(A)** Endogenous expression of PPARγ in a panel of lung cancer cells. **(B, C)** ORO lipid staining upon PPARγ activation in the lung panel. Five lung cancer cell lines involving H3255, A549, and Calu6 (B); H1993 and H2073 (C) were treated with 30 μM of pioglitazone or 10 μM of troglitazone for 5 days in the RPMI media supplemented with 5% complete serum (or normal serum (NS)) or 5% lipid-depleted serum (or charcoal stripped serum (CS)), followed by ORO staining to detect lipid droplet accumulation. Note that H1993 and H2073 are the paired-match metastatic and primary tumors cells established from the same patient and PPARγ positive and PPARγ negative, respectively. All images of lipid staining were quantitatively represented as shown in bar graphs (right column). **(D)** Metabolic profiling of fatty acids upon ligand activation of PPARγ. Lung cancer cells H3255 (PPARγ positive) and H2073 (PPARγ negative) were treated with 30 μM of pioglitazone for 5 days in the RPMI media supplemented with 5% CS, followed by free fatty acid extraction and HPLC analysis to profile lipid content. Values are mean ± S.E.M. Difference between vehicle and treated samples was analyzed using one-way ANOVA (Tukey’s post-tests) (B and C) or 2-tailed unpaired *t*-test (D). * *p* < 0.05; ^#^ *p* < 0.01; ^Δ^ *p* < 0.001.

Srivastava et al. recently reported that PPARγ inhibition of tumor growth is mediated by metabolic switch of glucose to fatty acid oxidation toward increasing ROS [13]. Also, given that PPARγ is a master regulator in lipid metabolism, we wondered how PPARγ-mediated lipid metabolism would be involved in regulating REDOX balance in cancer. To execute systemic approach, we first set a panel of lung cancer cells including randomly selected lung cancer cells H1770, H3255, A549, H2347, and Calu6, and a genetic pair-matched set of H1993 and H2073 (Figure 1A and Supplementary Figure 1A). Note that H2073 is a primary tumor cell line and H1993 is the pair-matched metastatic tumor cells derived from the same patient [14]. Using these various lung cancer cells, PPARγ-mediated intracellular lipid change was investigated upon TZD treatment. Interestingly, Oil-red O (ORO) staining showed significant increase of lipid droplets upon TZD treatment in PPARγ-positive H3255, A549, Calu6, and H1993 cells, but not in H2347 and PPARγ-negative H2073 cells (Figure 1B, 1C and Supplementary Figures 1B and 2). More interestingly, accumulation of lipid droplets occurs even in lipid depleted media upon TZD treatment, suggesting *de novo* lipid biosynthesis upon PPARγ activation (Figure 1B, 1C and Supplementary Figure 2). PPARγ-mediated *de novo* lipid synthesis reaches the maximum at day 4, maintained up to day 5, and reduced at day 6 after TZD treatment (Supplementary Figure 2). It is of intriguing to note that H2347 cells showed TZD- induced mitochondrial β-oxidation as well as endogenous biosynthesis of fatty acid simultaneously, which is confirmed by treatment of etomoxir, a chemical inhibitor specifically blocking mitochondrial β-oxidation, in a charcoal-stripped serum (Supplementary Figure 3). This was supported by TZD-induced genetic signature for lipid uptake, biosynthesis, and mitochondrial β-oxidation (Figure 3E). Consistently, the previous study mentioned above showed induced expression of genes involved in both fatty acid biosynthesis and mitochondrial β-oxidation in H2347 cells upon TZD treatment. Along with the genetic signature and lipid staining, metabolic profiling for free fatty acid revealed unsaturated free fatty acid, C16:1 and C20:5n3 in intracellular lipid biosynthesis upon TZD treatment, which only observed in H3255 but not in PPARγ-negative cells H2073 (Figure 1D). Moreover, consistent with ORO staining data, TZD treatment inhibits proliferation of H3255, A549, Calu6, H1993, and H2347 cells but shows no effect on H2073 cell growth, in both full media and in lipid-depleted media (Supplementary Figure 4). Taken together, these data clearly highlight the potent effect of PPARγ to modulate lipid metabolism, especially *de novo* lipid synthesis subsequently suppress cancer growth.

### PPARγ activation modulates lipid metabolism in a sumoylation-dependent manner

**Figure 2:**
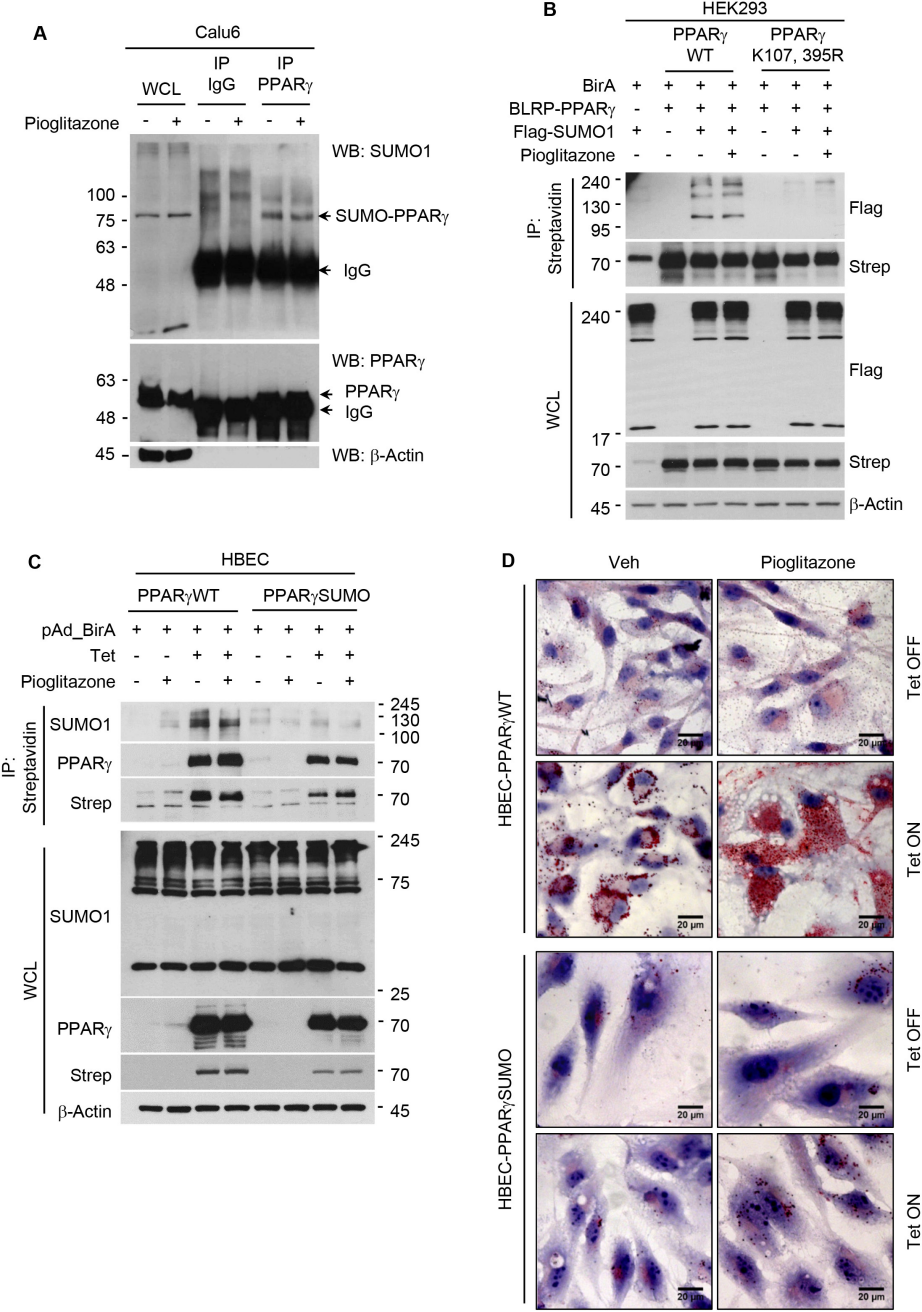

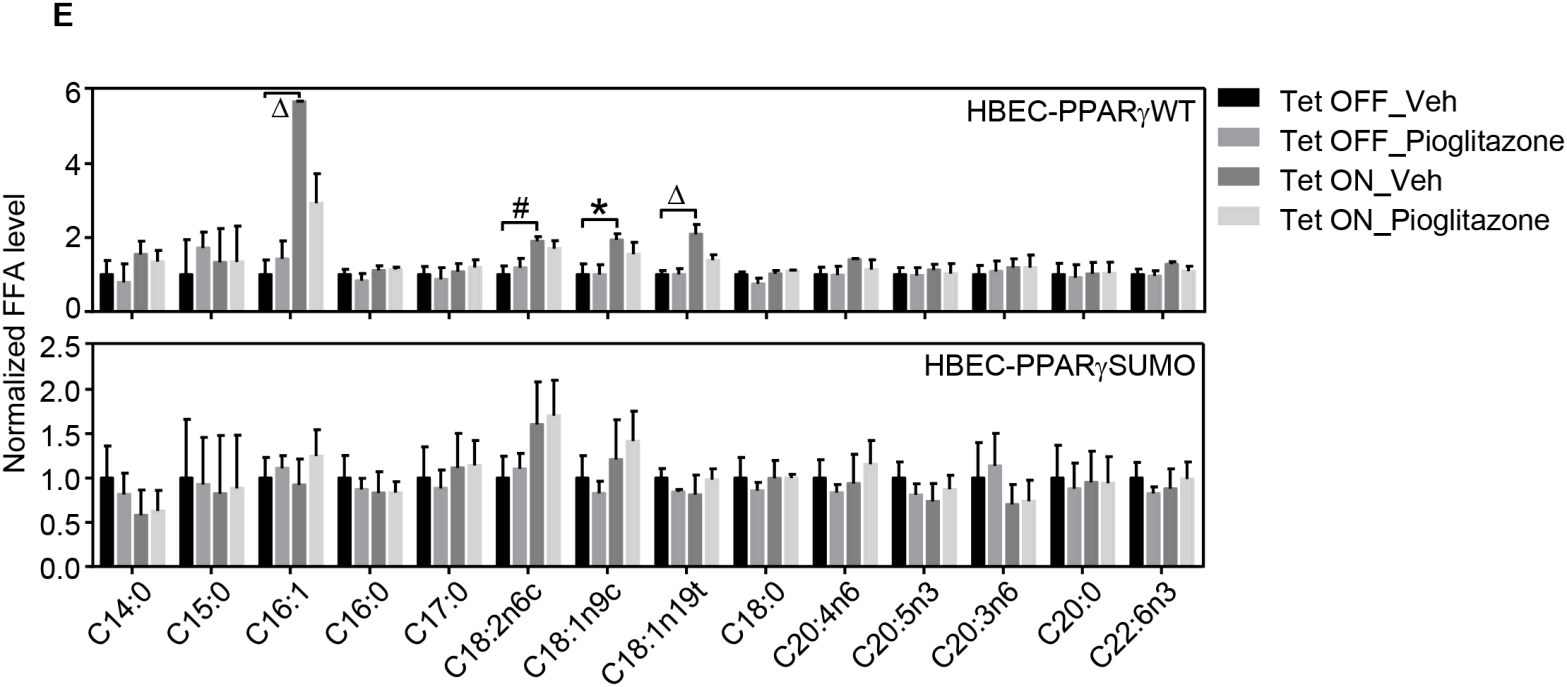
PPARγ activation induces *de novo* lipid synthesis in a sumoylation dependent manner **(A)** Sumoylation of endogenous PPARγ in Calu6. PPARγ immunoprecipitates from cell lysates treated with 30 μM of pioglitazone for 24 hours were subjected to western blot using indicated antibodies. **(B, C)** Sumoylation of PPARγWT or PPARγSUMO (K107, 395R). (B) HEK293 cells were co-transfected with plasmids expressing BirA, Flag-SUMO1, and BLRP-PPARγ WT or BLRP-PPARγ SUMO, and further treated with 3 μM of pioglitazone for 24 hours. Streptavidine (strep) immunoprecipitates were subjected to western blot using indicated antibodies. (C) Sumoylation of PPARγ in HBEC cells with stable expression of BLRP-PPARγ WT or BLRP-PPARγ SUMO upon tetracycline induction. Streptavidine immunoprecipitates from HBEC cells infected with pAd-BirA were subjected to western blot using indicated antibodies. **(D)** ORO lipid staining in HBEC cells. HBEC cells under tetracycline (Tet) ON or OFF conditions were treated with pioglitazone (3 μM) for 5 days in media containing 5% CS, followed by ORO staining to detect lipid droplet accumulation. **(E)** Metabolic profiling of fatty acids upon ligand activation of PPARγ. HBEC cells were treated with pioglitazone (3 μM) for 5 days, followed by free fatty acid extraction and HPLC analysis to profile lipid content. Values are mean ± S.E.M. Statistical analysis was performed using one-way ANOVA (Turkey’s post-tests). * *p* < 0.05; ^#^ *p* < 0.01; ^Δ^ *p* < 0.001. WCL, whole cell lysate; IP, immunoprecipitation; WB, western blot.

Since we recently reported that sumoylation of PPARγ is critical for PPARγ-mediated suppression of lung cancer growth as well as inhibition of inflammatory cyclooxygenase 2 expression, we here wanted to investigate if the same post-translational modification of PPARγ is also involved in cancer lipid metabolism [15]. We first identified sumoylation of endogenous and exogenous PPARγ in lung cancer Calu6 cells and HEK 293 transfected with PPARγ, respectively (Figure 2A and 2B). Note that PPARγ sumoylation occurs in PPARγWT, interestingly independent of ligand treatment, but not in PPARγSUMO harboring mutation at lysine 107 and lysine 395, while both PPARγWT and PPARγSUMO are confirmed to be transcriptionally functional using luciferase assay (Figure 2B and Supplementary Figure 5). For biological studies, we utilized human bronchial epithelial cell (HBEC) lines inducibly expressing PPARγWT or PPARγSUMO upon tetracycline treatment as previously described in detail [15]. Sumoylation of PPARγ in HBEC cells was confirmed by co-immunoprecipitation assay (Figure 2C). Using this well-characterized *in vitro* system, we next executed further independent biochemical assays to demonstrate biological correlation of the receptor sumoylation to lipid metabolism. Consistent with the observation in the lung cancer panel, ORO experiment showed TZD induction of lipid droplet accumulation in a PPARγ sumoylation-dependent manner, which was observed only in HBEC-PPARγWT but not in HBEC-PPARγSUMO (Figure 2D). In addition, metabolic profiling for intracellular fatty acid contents revealed that PPARγ activation in both complete serum as well as lipid-depleted conditions strongly induced unsaturated free fatty acid level, C16:1, C18:2n6c, C18:1n9c, and C18:1n19t, in HBEC-PPARγWT but not in HBEC-PPARγ-SUMO (Figure 2E). Taken together, this data suggest sumoylation of liganded PPARγ is critical for *de novo* lipid biosynthesis.

### PPARγ sumoylation induces gene expression involved in lipid metabolism

**Figure 3:**
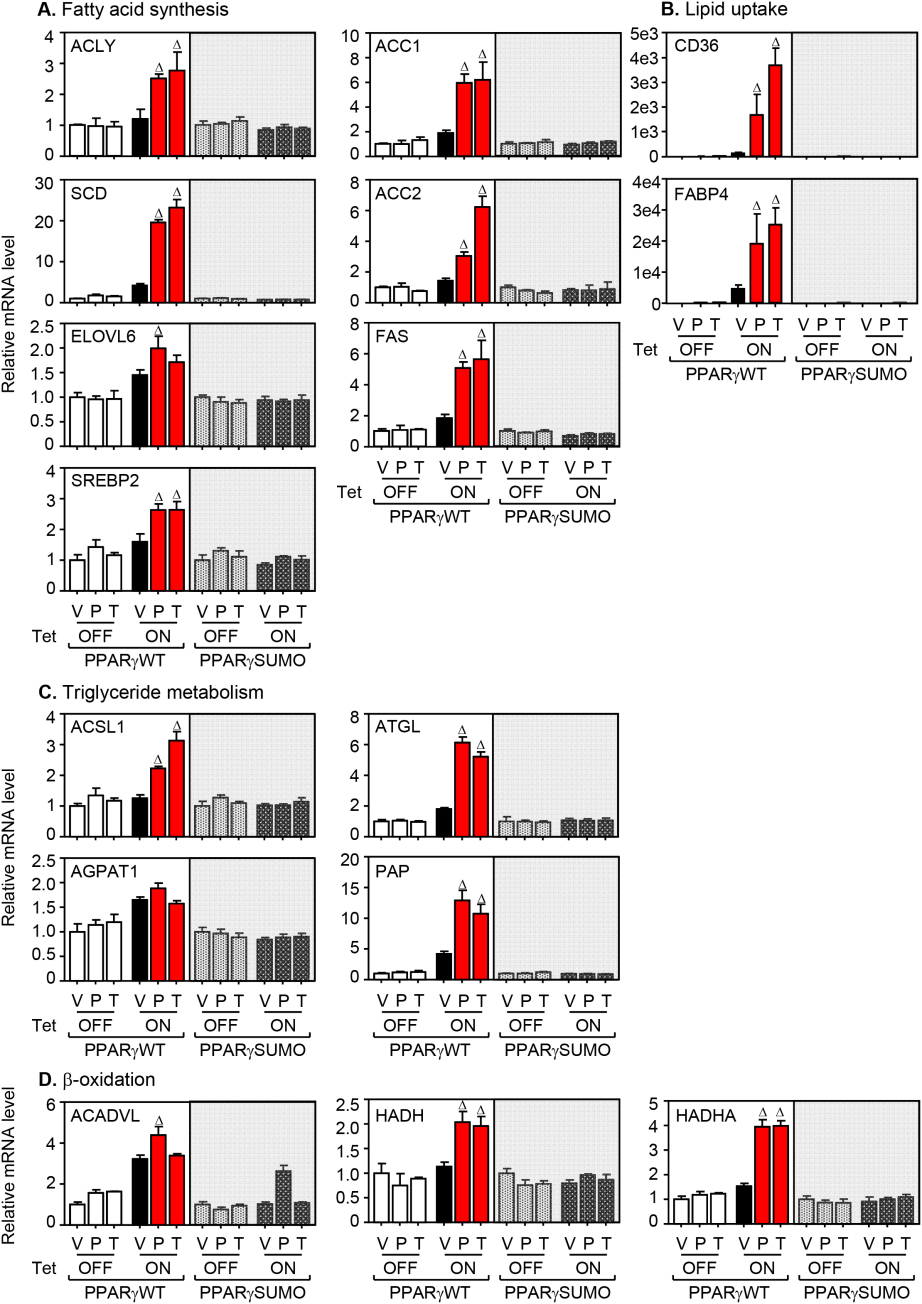

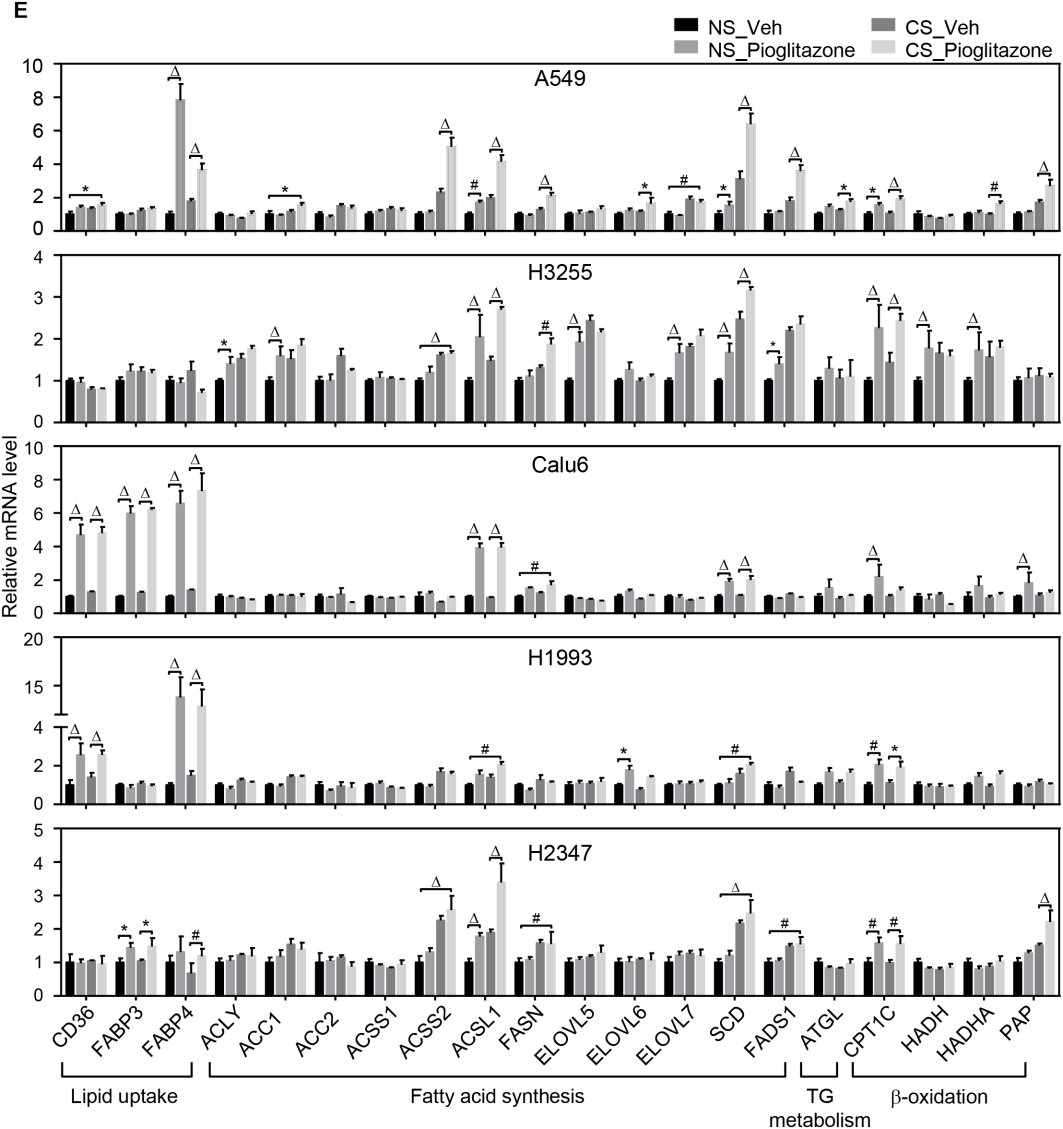
mRNA expression of genes involved in lipid metabolism upon PPARγ activation **(A-D)** HBEC cells treated with 3 μM of pioglitazone (P) or troglitazone (T) for 24 hours and followed by QPCR assay for mRNA expression of metabolic genes involved in lipid synthesis (A), lipid uptake (B), triglyceride metabolism (C) and β-oxidation (D). **(E)** Gene expression involved in lipid metabolism in various lung cell lines. Included are five lung cancer cell lines involving A549, H3255 Calu6, H1993, and H2347. During culture in the media supplemented with 5% NS or 5% CS, all cell lines were treated with pioglitazone 30 μM for 24 hours and followed by QPCR assay to profile mRNA expression of metabolic genes involved in lipid metabolism. Values are mean ± S.E.M. Statistical was analysis was performed using one-way ANOVA (Tukey’s post-tests). * *p* < 0.05; ^#^ *p* < 0.01; ^Δ^ *p* < 0.001.

Having demonstrated that sumoylation of liganded PPARγ increases intracellular lipid accumulation, we next wanted to know the expression profile of lipid metabolic genes in the HBECs and the same lung cancer panels upon TZD treatment under serum-supplemented or -depleted condition. Expression signature of twenty-two different metabolic genes was surveyed by quantitative real-time PCR assay using the corresponding primer sets (Supplementary Table 1). Included are 4 different subsets of genes involved in lipid metabolism: 1) ATP-citrate lyase (ACLY), acetyl-CoA carboxylase (ACC) 1, ACC2, acyl-CoA synthetase short-chain family member (ACSS) 1, ACSS2, stearoyl-CoA desaturase (SCD), elongation of long-chain fatty family member (ELOVL) 5, ELOVL6, sterol regulatory element-binding protein (SREBP), fatty acid desaturase (FADS) 1, and fatty acid synthase (FASN) for fatty acid biosynthesis; 2) cluster of differentiation 36 (CD36), fatty acid binding protein (FABP) 3, and FABP4 for lipid uptake; 3) acyl-CoA synthetase long-chain family (ACSL) 1, adipose triglyceride lipase (ATGL), 1-acylglycerol-3-phosphate acyltransferase alpha (AGPAT1), and phosphatidic acid phosphatase (PAP) for triglyceride metabolism; 4) carnitine palmitoyl transferase (CPT) 1c, acyl-CoA dehydrogenase, very long chain (ACADVL), hydroxyacyl-CoA dehydrogenase (HADH), and hydroxyacyl-CoA dehydrogenase/3-ketoacyl-CoA thiolase/enoyl-coA hydratase (HADHA) for β-oxidation. Consistent with ORO staining and metabolic profile of fatty acids, the genetic profiling showed that the expression of lipid metabolic genes was increased in HBEC-PPARγWT cells, but not in HBEC-PPARγSUMO cells upon TZD treatments (Figure 3A-3C). Interestingly, the HBEC-PPARγWT cells revealed simultaneous increase of gene expression involved in mitochondrial β-oxidation when treated with TZDs (Figure 3D). Likewise, TZD treatment significantly induced the expression of genes associated with lipid catabolism as well as both lipid uptake and biosynthesis in the lung cancer cells (Figure 3E). Overall, this data suggest that liganded PPARγ modulates the expression of subsets of genes involved in both lipid biosynthesis as well as mitochondrial β-oxidation, which occurs to be in a PPARγ-sumoylation manner.

### PPARγ activation regulates lipid metabolism *in vivo*

**Figure 4:**
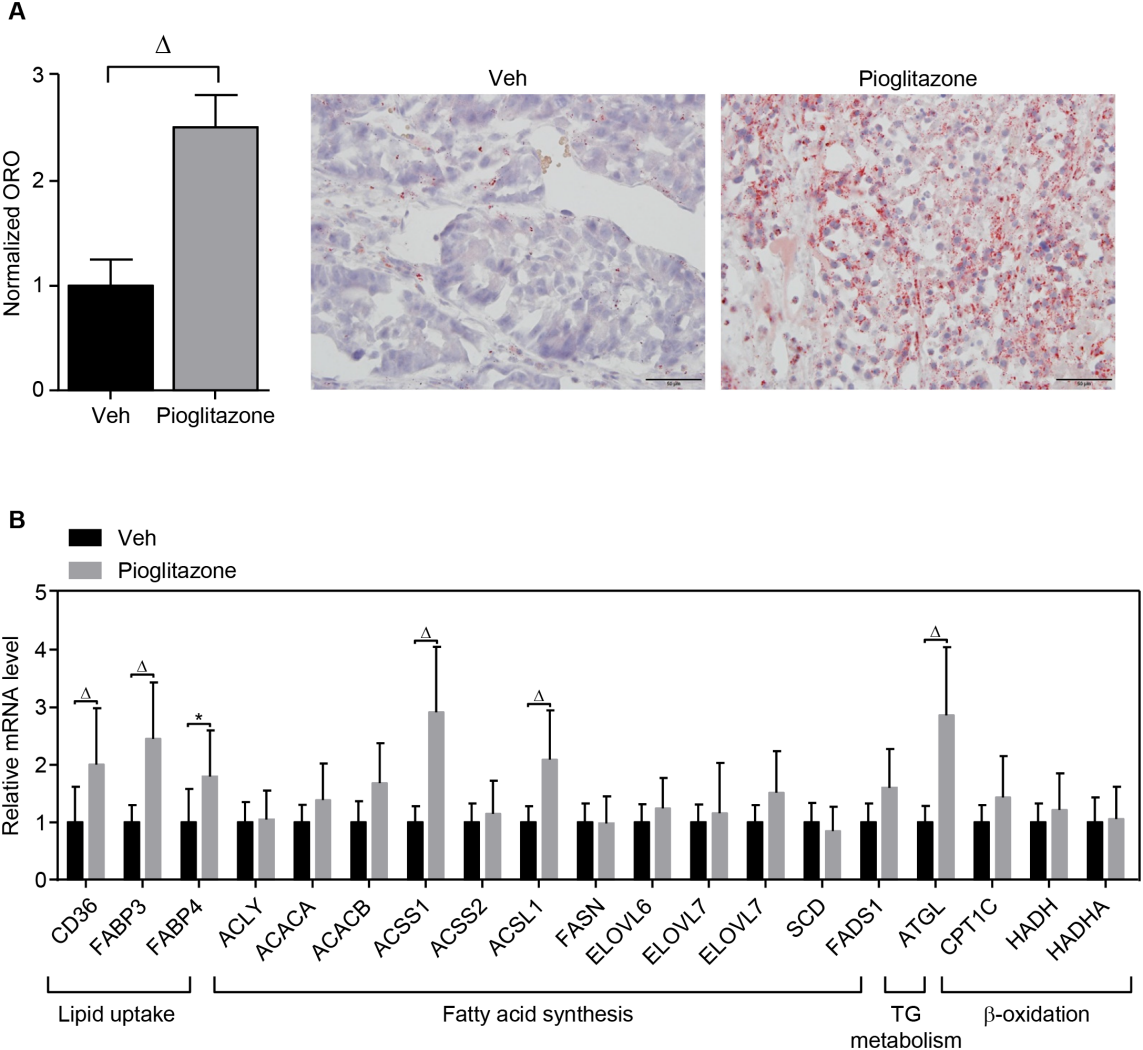
PPARγ regulation of lipid metabolism in the xenograft model *In vivo* xenograft tumor model was established by injecting PPARγ-positive A549 lung cancer cells into the flank of athymic nude mice. The xenografted mice were intraperitoneally injected with vehicle (*n* = 4) or pioglitazone 20mg/kg (*n* = 5) every other day for 3 weeks. At the end of the experiment, tumor tissues were isolated for ORO staining **(A)** and QPCR assay **(B)** for metabolic genes involved in lipid metabolism. ORO staining was performed in different tumor sections for each group (vehicle, *n* = 4; pioglitazone, *n* = 5). On each tumor section, pictures were taken from 10 separated regions. Representative pictures were shown. Values are mean ± S.E.M. 2-tailed unpaired *t*-test was applied for statistical analysis. * *p* < 0.05; ^#^ *p* < 0.01; ^Δ^ *p* < 0.001.

To confirm the *in vitro* study using *in vivo* model, the xenografted tumor model for A549 lung cancer was established in the flank region of athymic nude mice and followed by intraperitoneal administration of 20 mg/kg of pioglitazone or vehicle every other day as described in materials and methods. We found that pioglitazone treatment dramatically increased lipid accumulation in A549 tumor tissues (Figure 4A). Consistently, the expression of metabolic genes involved in lipid metabolism was increased in the pioglitazone-treated A549 xenograft tumor tissues (Figure 4B). In particular, the gene expression for lipid uptake and storage was induced in the xenograft model upon pioglitazone administration, suggesting that PPARγ activation might be more involved in systemic lipid mobilization for cancer growth.

### PPARγ activation disrupts REDOX balance in lung cancer cells

**Figure 5:**
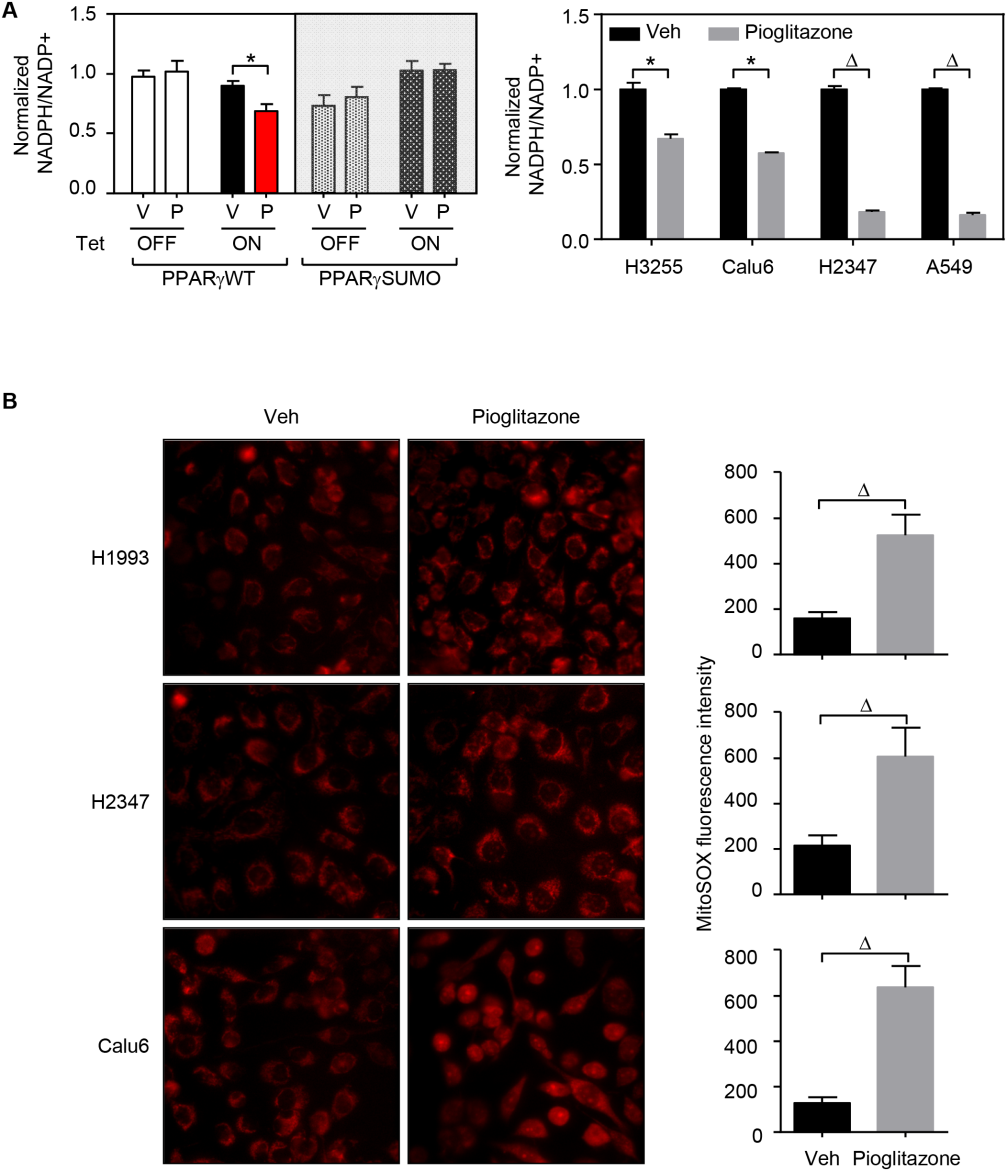
PPARγ activation disrupts REDOX balance in lung cancer **(A)** NADPH level upon pioglitazone treatment. HBEC cells and lung cancer cell lines (H3255, Calu6, H2347, and A549) were treated with 3μM or 30 μM of pioglitazone for 48 hours, respectively. The samples were then followed by measurement assay of NADPH level. **(B)** ROS level upon pioglitazone treatment. Three lung cancer cells (H1993, H2347, and Calu6) were treated with 30 μM of pioglitazone for 48 hours, followed by measurement assay of mitochondrial ROS level, mitoSOX. Mitochondrial ROS measurement was performed in three different coverslips for each group. On each coverslip, pictures were taken from 10 separated regions. Representative pictures were shown. Statistical analysis of One-way ANOVA (Tukey’s post-tests) or 2-tailed unpaired *t*-test was applied for HBEC sample or for lung cancer cells, respectively. * *p* < 0.05; ^#^ *p* < 0.01; ^Δ^ *p* < 0.001. Values are mean ± S.E.M.

Given that PPARγ activation evidently modulates lipid metabolism in lung cancer and generally known for inhibiting cancer cell proliferation, we wondered how the biochemical feature of the nuclear receptor would be mechanistically involved in the tumor suppressive function. While lipid metabolism is generally critical for tumor growth, a robust elevation of fatty acid synthesis might cause intracellular depletion of NADPH source, leading to tumor suppression [26, 27]. Indeed, treatment of TZDs results in significant decrease of intracellular NADPH levels in lung cancer cells (Figure 5A). Moreover, as NADPH is a critical cellular reducing power for regeneration of glutathione scavenging mitochondrial ROS that maintains optimal cellular REDOX balance [26], we measured mitochondrial ROS level under TZD treatment condition. Certainly, ligand-activation of PPARγ strongly induced mitochondrial ROS level in lung cancer cells (Figure 5B). Indeed, Srivastava et al., have previously reported that treatment with the antioxidant N-acetylcysteine (NAC) can rescue the increase in ROS levels upon pioglitazone treatment and mitigate the anti-tumorigenic effects of pioglitazone [13]. Consistently, we also confirmed that NAC treatment significantly rescues TZD-mediated cell growth inhibition in lung cancer cells A549 and H2347 (Supplementary Figure 6). Taken together, these data clearly suggest that, PPARγ disrupts REDOX balance by regulating cellular lipid metabolism which depletes NADPH by fatty acid synthesis and increases mitochondrial ROS from β-oxidation in lung cancer cells.

## DISCUSSION

Metabolic rewiring during oncogene-driven tumor progression is believed to be a crucial biochemical adaptation process for lung cancer initiation as well as progression, but also tumors once established become dependent on the reprogrammed metabolic pathways for maintaining the malignancy and aggressiveness [5]. Therefore, combined targeting oncogenic drivers and metabolic dependency such as bioenergetics, biosynthesis, and REDOX balance would be opted as a potential therapeutic approach for lung cancer in the clinical setting. Recently, diverse preclinical and clinical studies have been performed targeting glucose, glutamine, and lipid metabolism for ‘un-targetable’ cancers acquired on-target resistance. Amongst these, targeting lipid metabolism, in particular *de novo* fatty acid synthesis and β-oxidation, might be a prospective anticancer strategy that interferes cellular REDOX balance leading to cancer growth inhibition [28–31].

Nuclear receptor PPARγ is a ligand-activated transcription factor that plays diverse physiological roles in regulation of whole body glucose homeostasis, insulin sensitivity, and adipose tissue differentiation for lipid homeostasis [11, 32]. Therefore, one might reasonably think that PPARγ could also be involved in cancer metabolism, once the receptor expression being identified in the cancer cells. Given that PPARγ has been reported as a potential tumor suppressor in various types of cancers, it is of interest to ask if the metabolic function of PPARγ, in particular lipid metabolism, would have something to do with its tumor suppressive function in lung cancer, which was previously shown to be PPARγ-sumoylation dependent [15].

In this study, we explored how PPARγ regulates lipid metabolism and what is the underlying biochemical mechanism for its anti-proliferative function in lung cancer. In this regard, several biochemical and molecular approaches have been performed: 1) to investigate if PPARγ regulates lipid metabolism in lung cancer cells; 2) to elucidate the function of PPARγ sumoylation in regulating cancer lipid metabolism; and 3) to assess the correlation between PPARγ-mediated lipid metabolism and REDOX status upon TZD treatment.

To that end, we first confirmed PPARγ expression followed by biochemical function of the ligand-activated receptor in a subset of lung cancer cells. From the biochemical assay and metabolomics profiling, we notably identified that TZD-mediated PPARγ activation induces lipid uptake, mitochondrial β-oxidation, and *de novo* lipid biosynthesis. Metabolomics profiling revealed that TZD treatment increases unsaturated free fatty acid in the intracellular lipid droplets. It is interesting to note that different subsets of lung cancer cells may have evolved differentiated molecular adaptation for maintaining intracellular lipid metabolism upon TZD treatment. For instance, TZD treatment induces basal lipid uptake in a subset of lung cells while the other, like H3255 cells, shows no lipid uptake in serum supplemented condition. However, TZD-induced endogenous lipid biosynthesis commonly dominated mitochondrial β-oxidation in all lung cells, but opposite in H2347 cells, tested in this study.

Secondly, we demonstrated that PPARγ-sumoylation is evidently involved in lipid metabolism in lung cancer. Sumoylation of PPARγ has been previously reported in regulation of PPARγ transcriptional activity for anti-inflammation as well as tumor suppression [15, 23, 25]. Our study showed that TZDs induce lipid metabolic genes in a PPARγ-sumoylation dependent manner. Even though the mutation disrupting PPARγ sumoylation at lysine 107 and lysine 395 does not affect PPARγ general transcriptional activity (Supplementary Figure 5), it selectively inhibits PPARγ transcriptional activity to regulate subset of PPARγ target genes involved in lipid metabolism. Moreover, it turned out that PPARγ not only regulates genes for fatty acid biosynthesis, but also induces genes involved in triglyceride metabolism as well as mitochondrial β-oxidation. A previous study reported that PPARγ activation induces mitochondrial β-oxidation subsequently generating mitochondrial ROS, which results in growth inhibition of lung adenocarcinoma H2347 cells. However, from the analysis of gene expression data in more detail, we were able to identify that activation of PPARγ induces gene expression for both β-oxidation and endogenous lipid biosynthesis in H2347 cells, suggesting that PPARγ might modulate intracellular lipid homeostasis by balancing uptake, anabolic, and catabolic processes of lipid. It would be intriguing to ask a question of how the metabolic balance between catabolism and anabolism of intracellular lipid could be finely regulated by PPARγ.

Lastly, we showed that tumor suppressive function of PPARγ is clearly associated with regulating intracellular REDOX balance in lung cancer. As a well-known metabolic hallmark in cancer, maintaining certain level of intracellular NADPH is crucial for controlling biosynthesis as well as REDOX balance in cancer cells [26]. On the other hand, it has been reported that fatty acid synthesis accounts for the highest percentage of cellular NADPH consumption [33, 34]. Therefore, while lipid metabolism is generally critical for tumor growth, a robust elevation of fatty acid synthesis might cause intracellular depletion of NADPH source, leading to tumor suppression [26]. Indeed, a previous study has suggested that by inhibiting fatty acid synthesis, AMPK reserves cellular NADPH level in nutrient stress condition and thus promotes tumor growth [27]. In this regard, PPARγ-mediated lipid metabolism is believed to be critical to decrease intracellular reducing power NADPH since the receptor-mediated regulation of lipid metabolism for both anabolic lipid biosynthesis and catabolic β-oxidation pathways, eventually results in intracellular NADPH depletion. This subsequently leads to severe impair of REDOX balance and thus cancer suppression.

Taken together, this study provides an insight of insulin sensitizers-mediated cancer metabolism into understanding the prognostic benefit as well as therapeutic potential of the PPARγ in the cancer clinic.

## MATERIALS AND METHODS

### Cell culture and reagents

Lung cancer cells were cultured in RPMI 1640 (H2073, H3255, H2347, Calu6, and H1993) or DMEM media (H1299 and A549) supplemented with 5% FBS, 50 U/mL penicillin, and 50 U/mL streptomycin, at 37°C, 5% CO_2_ atmosphere. Cells were periodically tested for mycoplasma contamination. HBEC stable cell lines with inducible PPARγ expression were generated as previously reported [15]. Particularly, HBEC cells containing wild-type PPARγ or sumoylation mutant PPARγ (K107, 395R), HBEC-PPARγWT and HBEC-PPARγSUMO, are two tumorigenic clones in which biotin ligase recognition peptide (BLRP)-tagged PPARγWT or PPARγSUMO are tightly regulated upon tetracycline treatment. HBEC cells were cultured in RPMI 1640 supplemented with 10% FBS, 50 U/mL penicillin and 50 U/mL streptomycin. PPARγ agonists (pioglitazone and troglitazone) were purchased from Santa Cruz, tetracycline was from Sigma.

### Immunoblot analysis

Whole cell lysates and homogenized tumors were prepared in lysis buffer (150 mM NaCl, 1% triton X-100, 0.5% sodium deoxycholate, 0.1% SDS, 50 mM tris pH 8), containing protease inhibitor (Sigma) and phosphatase inhibitor (Sigma). Protein concentration was determined by BCA protein assay (Pierce) and protein lysates were then reduced and denatured by boiling for 5 minutes at 100°C in SDS-sample buffer (50 mM tris pH 6.8, 2% SDS, 0.02% bromophenol blue, 10% glycerol, 1% beta-mercapto ethanol). The lysates were then loaded on SDS-PAGE gels, subsequently transferred to nitrocellulose membrane (BioTrace). Transfer of proteins to membrane was checked by Ponceau staining. The membranes were blocked in 5% skim milk in tris buffer saline containing 0.1% tween 20 (TBST) for 1 hour at room temperature and then incubated with primary antibodies overnight at 4°C at manufacturer recommended concentration. The membranes were next washed in TBST and incubated with appropriate secondary antibodies at room temperature following manufacturer’s instructions for 1 hour. Finally, the membranes were washed in TBST and band images were acquired using X-ray film (Fuji), X-ray developer and fixer (Vivid). Primary antibodies recognizing PPARγ was purchased from Cell Signaling Technology (#2435). Antibody recognizing β-actin (Ab6276) was from Abcam. Secondary antibodies were anti-mouse IgG, HRP conjugated, from Abcam (ab6728) and anti-rabbit IgG, HRP conjugated, from Santa Cruz (sc-2030).

### Microarray data analysis

Microarray data were extracted from a GEO dataset (GSE accession number: GSE4824). Matrix 1.29 software was used for expression analysis of metabolic gene involved in lipid metabolism.

### Oil red O staining

Cells were seeded on coverslips and cultured in medium containing 5% complete serum (or normal serum (NS)) or 5% lipid-depleted serum (or charcoal stripped serum (CS)) in which non-polar compounds such as lipophilic materials were removed by charcoal or charcoal-dextran. After treated with drugs as desired, cells were fixed in 3.7% formaldehyde for 30 minutes, washed with water and then in 60% isopropanol. Cells were then stained with 0.5% ORO solution (Sigma) diluted with distilled water (ORO: water = 3 : 2) for 5 minutes. Subsequently, cells were fully washed with water, counterstained with hematoxylin and observed under microscopy. Every experiment was carried out in three different coverslips for each group. On each coverslip, pictures are taken from 10 separated regions.

### Sumoylation assay

HEK293 cells transfected with BLRP-tagged PPARγWT or PPARγSUMO (K107, 395R), biotin-protein ligase (BirA) and Flag-tagged SUMO1, or HBEC cells infected with BirA adenovirus were lysed in immunoprecipitation (IP) lysis buffer containing 10 mM tris-HCl pH 7.5, 150 mM NaCl, 10 mM phosphate buffer, 1% triton X-100, 20 mM N-ethylmaleimide (Sigma) and protease inhibitor cocktail (Roche). Cell lysates were immunoprecipitated overnight at 4°C with streptavidin resin beads (Thermo) and washed 5 times in IP washing buffer (phosphate-buffered saline with 0.5% NP40). Immunoprecipitates were resolved by SDS-PAGE and immunoblotted using anti-Streptavidin-HRP (Cell Signaling Technology, #3999) and anti-Flag-HRP antibodies (Sigma, #A8592).

For endogenous detection of sumoylated PPARγ, Calu6 cells treated with pioglitazone 30μM for 24 hours were harvested in IP lysis buffer, followed by incubation overnight at 4°C with SUMO1 antibody (#4940), IP with protein A/G-coupled agarose beads (Thermo) and 5 washes with IP washing buffer. Immunoprecipitates were then resolved by SDS-PAGE and immunoblotted using indicated antibodies.

### Luciferase assay

HEK293 cells were transfected with PPARγWT or PPARγSUMO (K107R and/or K395R) or control plasmids in combination with a luciferase reporter plasmid of PPAR response element (TK-PPRE3x-Luc) and renilla plasmid for normalization of transfection efficiency. Cells were then treated with vehicle or 3 μM of pioglitazone or troglitazone and assayed for luciferase activity.

### QPCR analysis

Total RNA was prepared from cells or homogenized tumor tissues using TRIzol reagent (Invitrogen). QPCR RT Master Mix (Toyobo) was then used to reverse-transcribe total RNA to cDNA. The mRNA expression for genes of interest was determined by QPCR in an ABI Prism 7900 HT Sequence Detection System (Applied Biosystems). Three replicates of each PCR reactions were carried out using SYBR green real-time PCR master mixes (Life Technologies). Data analysis was performed using delta-delta Ct method with 18S as the reference gene. Primer sequences were described in Supplementary Table 1.

### NADPH measurement

Cells washed with cold PBS and lysed with NADP/NADPH extraction buffer (20 mM nicotinamide, 20 mM NaHCO_3_, 100 mM Na_2_CO_3_). NADPH standards (0 to 10μM) were made fresh for every experiment from stock solution. Samples and standards are heat at 60°C for 30 minutes to destroy NADP^+^ for NADPH assay. Assay reaction includes 10 μL samples or standard with 80 μL cycling buffer (100 mM Tris-HCl pH 8, 0.5 mM MTT, 2 mM PES, 5 mM Na_4_EDTA, 1.3 IU/mL G6PD) and 20 μL 100 mM G6P. Absorbance is measured at 570 nM every 30 seconds for 10 minutes at 30°C. NADP+ is calculated as NADP^+^ = total NADP – NADPH.

### Mitochondrial ROS measurement

Mitochondrial superoxide was assessed in lived cells by MitoSOX probe. Cells were plated on cover slips and treated with pioglitazone 30 μM in 24 hours. Culture medium was removed, cells were incubated with MitoSOX at 5 μM final concentration in KRB solution in 30 minutes at room temperature. Cells were then analysed by confocal microscope. The experiment was performed in 3 different cover slips for each group. On each cover slip, pictures were taken from 10 separated regions which has 30 - 40 cells. Metamorph was used to quantitate fluorescence intensity.

### Metabolomics profiling

Sample preparation for polar metabolite and lipid measurement: Cells, washed with cold PBS and water, are harvested in cold methanol/H2O (80/20, v/v), followed by adding internal standard solutions (Ceramide, DAG, PLS, Glutamine, Malonyl IS). 1.5 mL collected supernatant after centrifugation is added with 1.8 mL H_2_O and 0.96 mL chloroform, mix well. Aqueous layer and organic layer are separated by centrifugation. The samples are reconstituted with acetonitrile/H2O (50/50, v/v), for polar metabolites, or with methanol, for non-polar lipids and metabolically profiled by HPLC.

### Xenograft experiment

All animal experiments were reviewed and approved by the Institutional Animal Care and Use Committee (IACUC) of Yonsei University (Wonju Campus), IACUC approval number YWC-140417-1.

For A549 xenografts, 5x10^6^ cells were subcutaneously implanted into the right flanks of 6-week old female Balb/c nude mice. Treatment started when tumors reached ∼ 100 mm^3^. Xenografted nude mice were divided randomly into two groups of six mice each. Mice were treated with either vehicle or 20 mg/kg of pioglitazone intraperitoneally every other day for 14 days. At the end of the experiment, tumor tissues were isolated for ORO staining and QPCR assay.

### Statistical analysis

Statistical analysis and graphing are performed using Microsoft Excel 2013 and Graphpad Prism 6.0 software. The data were presented as mean ± SEM. Statistical significance was determined by one-way ANOVA (Tukey’s post-tests) (Figures 1B, 1C, 2E, 3, and 5A-left) or by 2-tailed unpaired *t* test (Figures 1D, 4, 5A-right, 5B and Supplementary Figures 4 and 6). * *p* < 0.05; ^#^ *p* < 0.01; ^Δ^ *p* < 0.001.

## SUPPLEMENTARY MATERIALS FIGURES AND TABLE




