# Correction: PPARγ sumoylation-mediated lipid accumulation in lung cancer

**DOI:** 10.18632/oncotarget.26710

**Published:** 2019-02-15

**Authors:** Ai N.H. Phan, Vu T.A. Vo, Tuyen N.M. Hua, Min-Kyu Kim, Se-Young Jo, Jong-Whan Choi, Hyun-Won Kim, Jaekyoung Son, Young-Ah Suh, Yangsik Jeong

**Affiliations:** ^1^ Department of Biochemistry, Wonju College of Medicine, Yonsei University, Wonju, Gangwon-do, Republic of Korea; ^2^ Department of Global Medical Science, Institute of Lifestyle Medicine, Wonju College of Medicine, Yonsei University, Wonju, Gangwon-do, Republic of Korea; ^3^ Asan Institute for Life Sciences, Asan Medical Center, College of Medicine, University of Ulsan, Seoul, Songpa-gu, Republic of Korea

**This article has been corrected:** The correct Grant Support information is given below:

**GRANT SUPPORT**

This study was financially supported by the Basic Science Research Program through the National Research Foundation of Korea funded by the Ministry of Education, Science and Technology (NRF-2016R1D1A3B03930581 to YS Jeong), Medical Research Center Program (2017R1A5A2015369), Yonsei University Future-leading Research Initiative of 2015 (RMS2-2015-22-0094), JJ-2017-1 to YS Jeong, and a grant of the Korea Health Technology R&D Project through the Korea Health Industry Development Institute (KHIDI), funded by the Ministry of Health & Welfare, Republic of Korea (HI17C0039).

Original article: Oncotarget. 2017; 8:82491-82505. https://doi.org/10.18632/oncotarget.19700

