# Supplementary material for: PPARγ sumoylation-mediated lipid accumulation in lung cancer
Source: Oncotarget. 2017 Jul 31;8(47):82491–505. doi: 10.18632/oncotarget.19700 (PMC5669906; doi:10.18632/oncotarget.19700)
Supplement: Supplementary file 1 [file oncotarget-08-82491-s001.pdf]

PPARγ sumoylation-mediated lipid accumulation in lung cancer

SUPPLEMENTARY MATERIALS

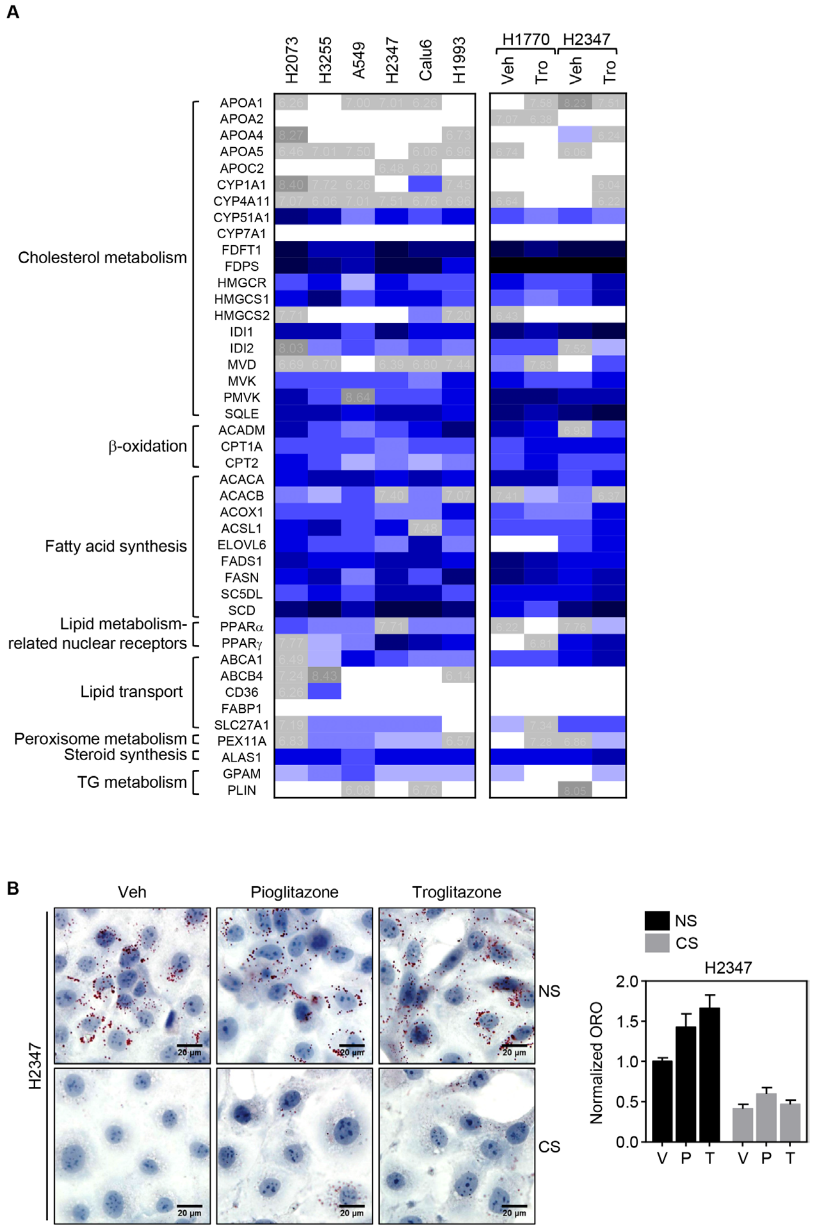

Supplementary Figure 1: Genetic signature of metabolic genes relevant to lipid metabolism in a subset of lung cancer cells. (A) Microarray data represent genetic signature of metabolic genes relevant to lipid metabolism in lung cancer cells including H2073, H3255, A549, H2347, Calu6, and H1993 (left panel). H1770 and H2347 cells, PPARγ-negative and –positive, respectively, were treated with troglitazone for 20 hours and followed by microarray experiment (right panel). Microarray data were extracted from a GEO dataset (GSE accession number: GSE4824). PPARγ activation induced expression of metabolic genes associated with cholesterol metabolism (APOC2, CYP1A1, CYP4A11, CYP7A1, HMGCR, HMGCS1, MVD, MVK, and SQLE), β-oxidation (ACADM), fatty acid synthesis (ACOX1 and SCD), lipid transport (FABP1), and triglyceride metabolism (GPAM). **(B)** ORO lipid staining upon PPARγ activation in lung cancer cells treated with 30 μM of pioglitazone or 10 μM of troglitazone for 5 days in the RPMI media supplemented with 5% complete serum (or normal serum (NS)) or 5% lipid-depleted serum (or charcoal stripped serum (CS)). All images of lipid staining were quantitatively represented as shown in bar graphs (right column). Values are mean ± S.E.M. Difference between vehicle and treated samples was analyzed using one-way ANOVA (Tukey's post-tests). * $p < 0.05$; $^{\#}$ $p < 0.01$; $^{\Delta}$ $p < 0.001$.

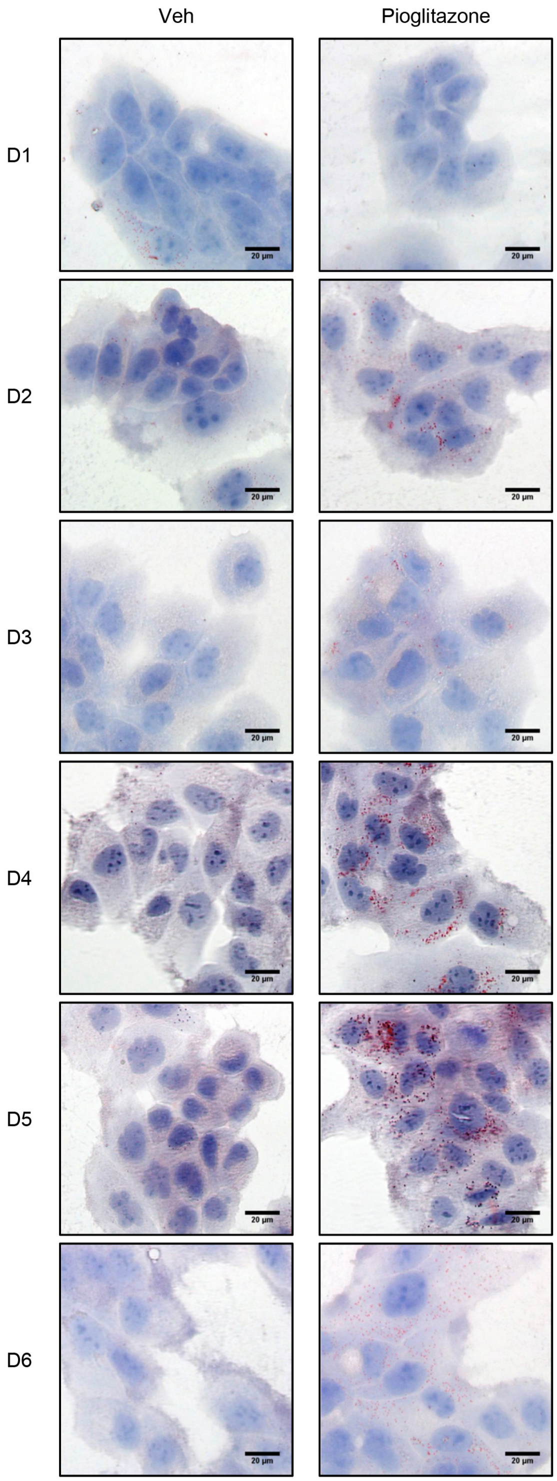

Supplementary Figure 2: TZD treatment induces *de novo* lipid synthesis in a time-dependent manner. Lung cancer cells H3255, cultured in 5% CS, were treated with pioglitazone 30 μM for 6 days in a time-dependent manner and followed by ORO staining to detect lipid droplet accumulation.

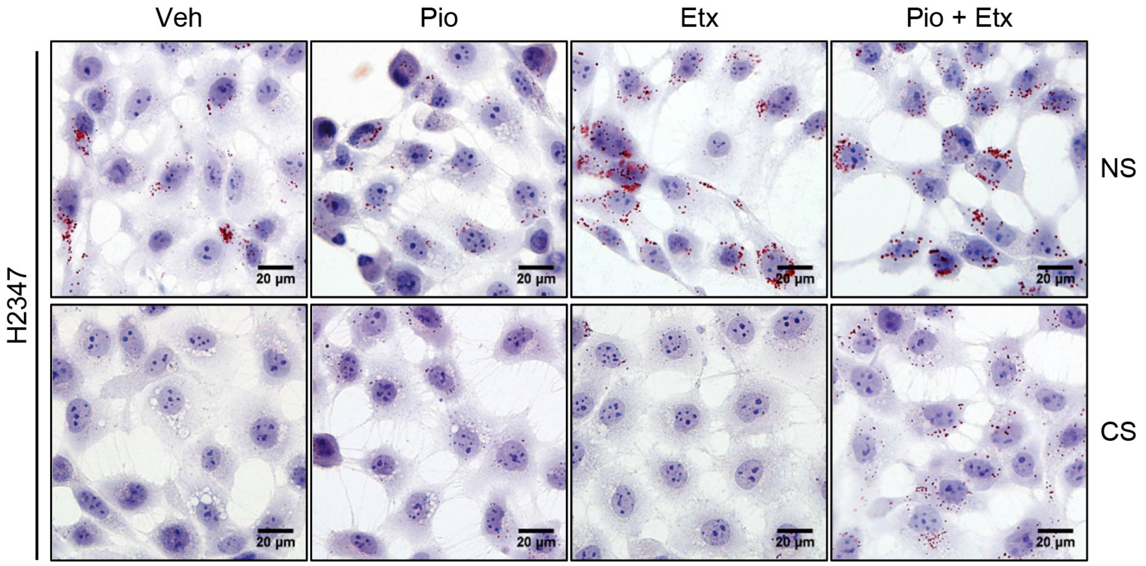

Supplementary Figure 3: PPARγ activation induces *de novo* lipid synthesis and β-oxidation in lung cancer cells. H2347 cells treated with pioglitazone (30 μM) and/or etomoxir (100 μM) for 4 days in media supplemented with 5% NS or 5% CS, and followed by ORO staining to detect lipid droplet accumulation.

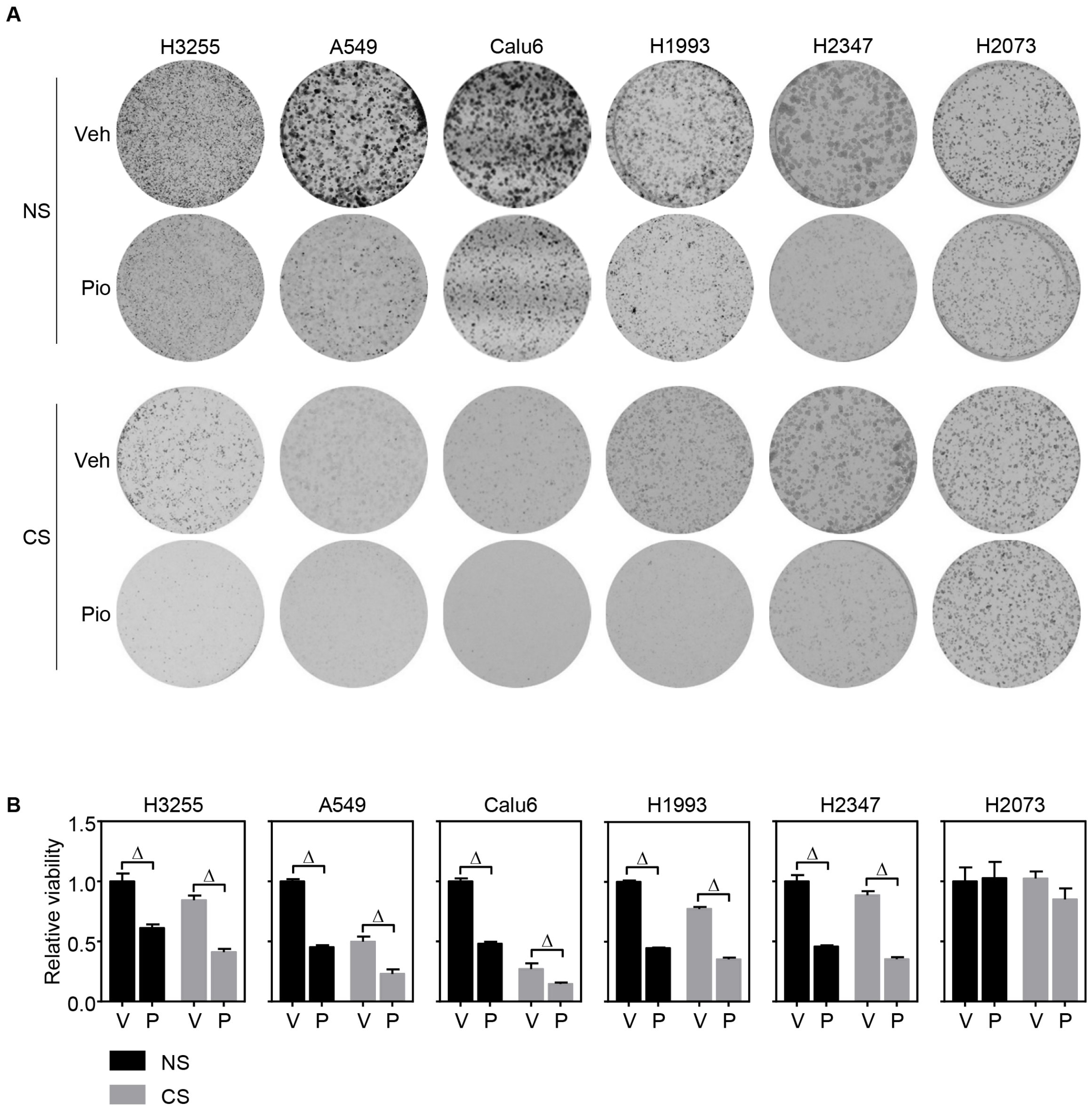

Supplementary Figure 4: Pioglitazone treatment inhibits lung cancer cell growth. Colony formation assay **(A)** and MTT assay **(B)** in six lung cancer cell lines involving H3255, A549, Calu6, H1993, H2347, and H2073 treated with 50 µM of pioglitazone (Pio) for 10 days (colony formation assay) or for 5 days (MTT assay) in media supplemented with 5% complete serum (or normal serum (NS)) or 5% lipid-depleted serum (or charcoal stripped serum (CS)). Values are mean ± S.E.M. Difference between vehicle and treated samples was analyzed using one-way ANOVA (Tukey's post-tests). * $p < 0.05$; $^{\#}$ $p < 0.01$; $^{\Delta}$ $p < 0.001$.

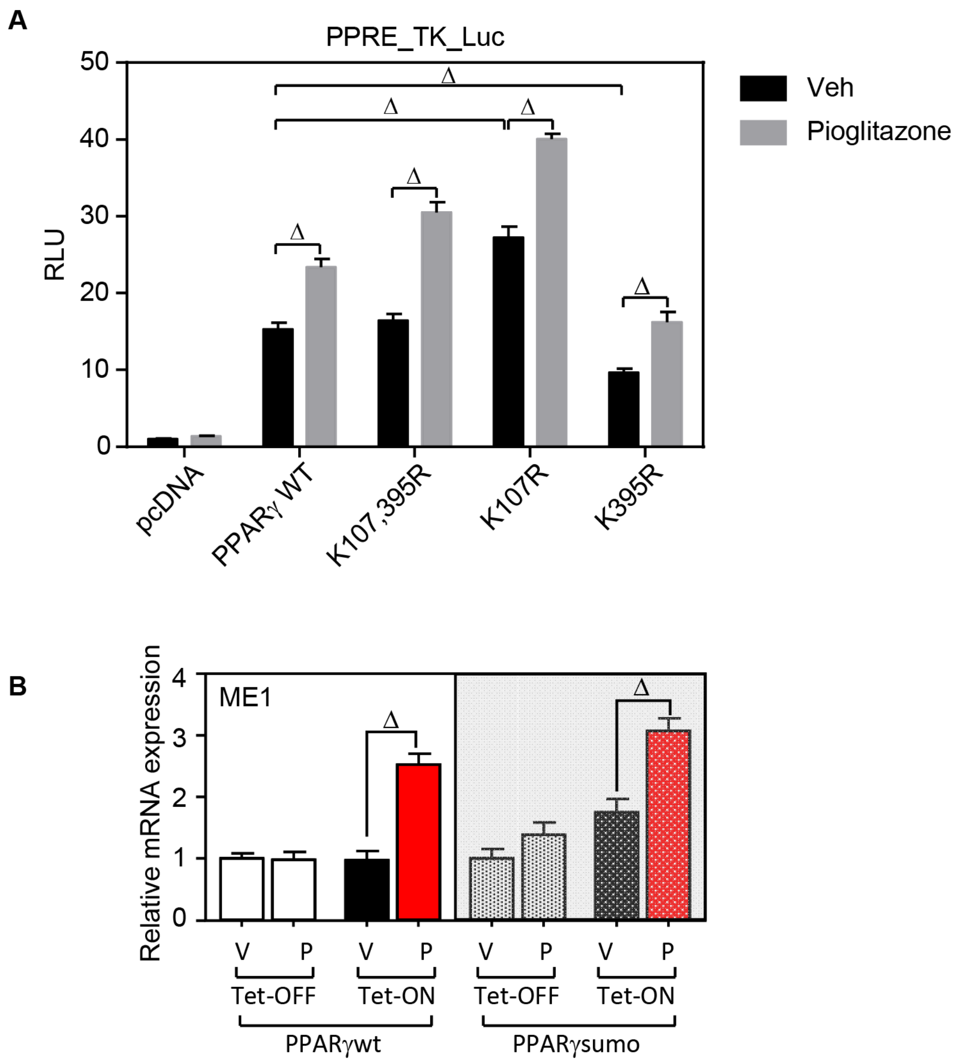

Supplementary Figure 5: Identification of various PPARγ constructs for transcriptional activity. (A) Transcriptional activity of PPARγWT and PPARγSUMO in HEK293 cells. Cells were co-transfected with PPARγ and PPRE-TK-Luc constructs, followed by measuring luciferase activity upon 3 μM of pioglitazone treatment for 24 hours. Note that PPRE-TK-luc construct involves luciferase gene under the control of thymidine kinase promoter with PPAR response element. **(B)** HBEC cells treated with 3 μM of pioglitazone (P) for 24 hours and followed by QPCR assay for mRNA expression of mallic enzyme 1 (ME1). Values are mean ± S.E.M. Data was statistically analyzed using one-way ANOVA (Tukey's post-tests). * $p < 0.05$; $^{\#} p < 0.01$; $^{\Delta} p < 0.001$.

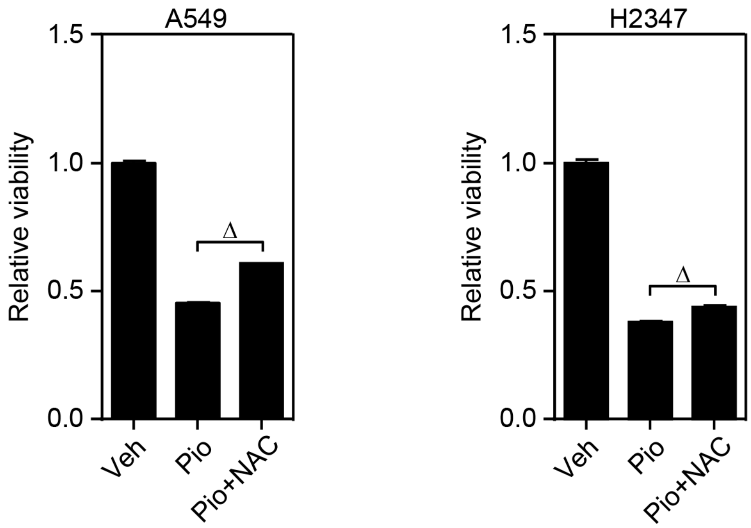

Supplementary Figure 6: N-acetyl cysteine treatment rescues pioglitazone-induced cell growth inhibition in lung cancer. MTT assay in lung cancer cells A549, and H2347 treated with 50 µM of pioglitazone and/or 5mM of N-acetyl cysteine (NAC) for 2 days in media supplemented with 5% complete serum (or normal serum (NS)) or 5% lipid-depleted serum (or charcoal stripped serum (CS)). Values are mean ± S.E.M. Difference between vehicle and treated samples was analyzed using one-way ANOVA (Tukey's post-tests). * $p < 0.05$; $^{\#} p < 0.01$; $^{\Delta} p < 0.001$.

Supplementary Table 1: QPCR primer sequences of genes involved in lipid metabolism

See Supplementary File 1
