# Supplementary material for: PPARγ sumoylation-mediated lipid accumulation in lung cancer
Source: Oncotarget. 2017 Jul 31;8(47):82491–505. doi: 10.18632/oncotarget.19700 (PMC5669906; doi:10.18632/oncotarget.19700)
Supplement: Supplementary file 2 [file oncotarget-08-82491-s002.docx]

**Supplementary Table 1: QPCR primer sequences of genes involved in lipid metabolism**

| **Gene Name** | **Gene Bank Accession number** | **5’-3’ Sequence** | | **Size (bp)** |
| --- | --- | --- | --- | --- |
| 18S | NR_003286 | F: | ACCGCAGCTAGGAATAATGGA | 63 |
|  |  | R: | GCCTCAGTTCCGAAAACCA |  |
| ACADVL | NM_000018 | F: | CTTTGCAACACCCAGTACGC | 109 |
|  |  | R: | GCAGGATGCCTTTGAAACCG |  |
| ACC1 | NM_198834 | F: | TGT TAC TCG CTT TGG GGG AAA | 155 |
|  |  | R: | CAG GTG TGA CCA TGA CAA CG |  |
| ACC2 | NM_001093 | F: | GGCTGGGAGCAGCTACGCTG | 103 |
|  |  | R: | CACGGCACCTGGACGCTTGA |  |
| ACLY | NM_001096 | F: | TCA CTC CTG ACA CAG ACT GG | 100 |
|  |  | R: | CCA CGA CGT TTG ATC AGC TG |  |
| ACSL1 | NM_001995 | F: | GCAGCGGCATCATCAGAAAC | 70 |
|  |  | R: | CCCAGGCTCGACTGTACTTT |  |
| ACSS1 | NM_032501 | F: | GGCTCACAGGACAGACAACA | 72 |
|  |  | R: | TCCTTGGCCATTTCCTGCTC |  |
| ACSS2 | NM_ 001076552 | F: | GTGTTCAGCCCCAGATCTCA | 80 |
|  |  | R: | ACACTCATCCCCTGCCTCTT |  |
| AGPAT1 | NM_006411 | F: | CAGGTTCCCATTGTCCCCAT | 84 |
|  |  | R: | ACATTGTCCCGAGGTGAAGC |  |
| ATGL | NM_020376 | F: | GCTCATCCAGGCCAATGTCT | 122 |
|  |  | R: | GCTCATAGAGTGGCAGGTTG |  |
| CD36 | NM_ 001001548.2 | F: | CGGCTGCAGGTCAACCTATT | 115 |
|  |  | R: | CACCAATGGTCCCAGTCTCA |  |
| CPT1C | NM_001876 | F: | CGG CCG ATG TTA CGA CAG GT | 190 |
|  |  | R: | GAT GTC GCC TTT GCA GTG CC |  |
| ECHS1 | NM_004092 | F: | GCCTCGGGTGCTAACTTTGA | 76 |
|  |  | R: | TCAGTTGGATCAACCCCACG |  |
| ELOVL5 | NM_ 021814.4 | F: | TGTTCTGTGAGTTAGTAACAGGAG | 111 |
|  |  | R: | ACCACCAGAGGACACGGATA |  |
| ELOVL6 | NM_024090 | F: | CAAAGCACCCGAACTAGGAG | 92 |
|  |  | R: | GGAGCACAGTGATGTGGTGA |  |
| ELOVL7 | NM_ 001297618.1 | F: | GCAATGATAACTTTGTGATGTCTGG | 106 |
|  |  | R: | CAGGTACGTGCCATCCTCAA |  |
| FABP3 | NM_ 001320996.1 | F: | ACCTGCAGAAATGGGACGG | 81 |
|  |  | R: | CGTGGGTGAGTGTCAGGATGA |  |
| FABP4 | NM_ 001442.2 | F: | ATGGGGGTGTCCTGGTACAT | 95 |
|  |  | R: | GACGCATTCCACCACCAGTTT |  |
| FASN | NM_004104 | F: | TCTTCGGAGTCCACCCCAAGC | 136 |
|  |  | R: | ACCCAGACGCCAGTGTGTGTT |  |
| FADS1 | NM_ 013402.4 | F: | GTGGCTAGTGATCGACCGTAA | 117 |
|  |  | R: | GTGGCTAGTGATCGACCGTAA |  |
| HADH | NM_  001184705 | F: | TGCAGAAAACCCTAAGGCCG | 102 |
|  |  | R: | ACCAAGTCTGTGCTGTGGAC |  |

| HADHA | NM_000182 | F: | TGGTTAAGGATGGACCTGGC | 103 |
| --- | --- | --- | --- | --- |
|  |  | R: | TCCAGCTTCTTCGGGTCAAC |  |
| PAP | NM_  001261427 | F: | CATGAGCAGAGTGCAGACCA | 73 |
|  |  | R: | GAGCTCCTTCACGGTGACAA |  |
| SCD | NM_005063 | F: | GAG CCC TGT ATG GGA TCA CT | 111 |
|  |  | R: | GAC GAT GAG CTC CTG CTG TT |  |
| SREBF2 | NM_004599 | F: | CAACATTCAGCACCACTCCG | 122 |
|  |  | R: | TACCTGGGAGGATGTCACCA |  |

F, forward; R, reverse; bp, base pairs

18S, 18S ribosomal RNA; ACADVL, acyl-CoA dehydrogenase very long chain; ACC1/2, acetyl-CoA carboxylase 1/2, ACLY, ATP citrate lyase; ACSL1, acyl-CoA synthetase long-chain family member 1; ACSS1/2, acyl-CoA synthetase short-chain family member 1/2; AGPAT1, 1-acylglycerol-3-phosphate O-acyltransferase 1; ATGL, adipose triglyceride lipase; CD36, cluster of differentiation 36; CPT1c, carnitine palmitoyl transferase; ECHS1, enoyl-CoA hydratase short chain 1; ELOVL5/6/7, fatty acid elongase 5/6/7; FABP3/4, fatty acid binding protein 3/4; FADS1, fatty acid desaturase; FASN, fatty acid synthase; HADH, hydroxyacyl-CoA dehydrogenase; HADHA, 3-ketoacyl-CoA thiolase; PAP, phosphatidic acid phosphohydrolase; SCD, stearoyl-CoA desaturase; SREBF2, sterol regulatory element binding protein 2
